# Improving Net Photosynthetic Rate and Rooting Depth of Grapevines Through a Novel Irrigation Strategy in a Semi-Arid Climate

**DOI:** 10.3389/fpls.2020.575303

**Published:** 2020-08-27

**Authors:** Xiaochi Ma, Pete W. Jacoby, Karen A. Sanguinet

**Affiliations:** ^1^Department of Crop and Soil Sciences, Washington State University, Pullman, WA, United States; ^2^Department of Plant Sciences, University of California – Davis, Davis, CA, United States

**Keywords:** direct root-zone irrigation, drought, photosynthesis, leaf gas exchange, root development, *Vitis vinifera*, minirhizotron, water management

## Abstract

Direct root-zone irrigation (DRZ) is a novel subsurface irrigation strategy initially tested in vineyards for economizing water and securing grape production in arid regions with unstable climatic patterns. However, studies are lacking on the responses of grapevine leaf carbon assimilation and deep rooting patterns to the novel irrigation strategy, which are essential for optimizing grapevine growth and alleviating extreme water stress during periods of heat and drought. Thus, a two-year field study was conducted in a commercial vineyard of Cabernet Sauvignon (*Vitis vinifera* L.) under a semi-arid climate in Washington, USA to compare the differences in leaf gas exchange and root distribution along the 0–160 cm soil profile, combined with measurements of specific leaf area and total carbon and nitrogen content in leaves and shoots to compare DRZ and traditional surface drip irrigation (SD) under three watering regimes. Compared to SD, significantly higher rates of net CO_2_ assimilation, stomatal conductance and transpiration in leaves, which positively correlated to midday stem water potential, were found in grapevines irrigated through DRZ in both years. Meanwhile, DRZ reduced total root number by 50–60% and root length density (RLD) by 30–40% in the upper 60 cm soil at high (0.75–0.80 crop evapotranspiration) and moderate (0.60–0.65 crop evapotranspiration) irrigation rates, but no significant differences were found at low (0.45–0.50 crop evapotranspiration) irrigation rate between DRZ and SD. Higher root number and RLD were detected under DRZ within 60–160 cm soil depths, accompanied by a decreased ratio of total carbon to nitrogen content in leaves with slightly increased specific leaf area. Decreased rainfall and increased temperature in 2018 possibly amplified the positive effects of DRZ. Our study indicates that grapevines under DRZ could develop deeper roots for water uptake, which helps ameliorate water stress and improve the photosynthetic rate as well as enhance grapevine adaptation to semi-arid climates.

## Introduction

Grapevines (*Vitis* spp.) are one of the most important horticultural crops worldwide in terms of economic and social values. In many wine-growing regions, efficient water management in vineyards helps regulate vegetative growth of grapevines and optimize the balance between yield and berry quality (Bernardo et al., 2018). For arid regions where the limited precipitation hardly supports grapevine growth, irrigation plays a significant role in offsetting the water deficit (Alexandratos and Bruinsma, 2012; Fraga et al., 2018; Malek et al., 2018). In contrast, excessive and highly localized irrigation leads to soil hypoxia and salinity, excessive leaching, and increased energy use for pumping, which might also cause adverse effects on grapevine growth and production, groundwater contamination and a rapid decline in groundwater levels (Drew, 1997; Scanlon et al., 2012; Kisekka et al., 2019; Zhang et al., 2020). Moreover, unstable climate patterns and increased demand of agricultural water for food production intensify the pressure on global water resources (Mbow and Rosenzweig, 2019). Thus, development of efficient irrigation strategies is necessary to sustain viticulture and improve water productivity, achieving “more crop per drop” (Davies and Bennett, 2015; Costa et al., 2016).

Compared to surface irrigation systems, the application of deficit irrigation through regulated subsurface micro-irrigation systems could be a more efficient means to regulate grapevine growth, while enhancing crop water use efficiency (WUE_c_, yield per unit area per unit of applied water) and sustaining the vineyard management (e.g. reduce evapotranspiration, restrict water availability for weed growth), especially in arid areas with limited water supply (Ayars et al., 2015; Ma et al., 2020). With upgraded irrigation equipment and scheduling tools in recent decades, the benefits have been demonstrated through studies both on annual row crops (Bhattarai et al., 2008; Zaccaria et al., 2017; Murley et al., 2018) and woody perennial crops (Zhang et al., 2017; Martínez-Gimeno et al., 2018; Paris et al., 2018; Pisciotta et al., 2018). However, additional improvements in subsurface irrigation systems, such as easier access for belowground maintenance and convenient adjustments to water delivery depth, are required for maximizing the benefits of the investment, since the initial costs for subsurface irrigation are usually higher than those for traditional surface irrigation systems (Payero et al., 2005; Lamm et al., 2012; Ma et al., 2020).

Direct root-zone irrigation (DRZ) is a novel subsurface micro-irrigation system which was initially tested in vineyards and showed promise for improving WUE_c_ and sustaining grape yield and quality in a semi-arid climate (Jacoby and Ma, 2018). Compared to traditional irrigation systems, DRZ flexibly adjusts the installation position and water delivery depth, concurrently providing easier access for belowground system maintenance (Ma et al., 2019). In a scenario of climatic change, grapevines have a high demand for supplemental irrigation especially in areas with seasonal drought (Bonada et al., 2015; Bernardo et al., 2018; Douthe et al., 2018). However, more details regarding the physiological performance of grapevines under DRZ are needed to finely tune its application in vineyards and provide empirical evidence to avoid detrimental effects on grapevine growth from improper water deficit that is regulated by irrigation.

The root system is indispensable for plant growth and survival owing to its function in water and nutrient uptake (Volder et al., 2004; Osmont et al., 2007; Volder et al., 2009), while woody portions of the root system provide structure support for aboveground growth (Comas et al., 2010). Irrigation significantly affects root growth especially in arid regions as it influences soil water availability (Sánchez-Blanco et al., 2019), which also influences plant water status and leaf gas exchange (Koundouras et al., 2008; Ko and Piccinni, 2009). Previous studies showed that DRZ restricts shallow root growth to potentially minimize the negative influence of fluctuations in precipitation and soil moisture within the top soil profile (Ma et al., 2020). Additionally, the deep root system of grapevines is vital for sustainable growth due to its potential to support grapevine throughout periods of drought and heat during the summer months (Savi et al., 2018). However, the effects of DRZ and other subsurface irrigation strategies on root growth in the deep soil profile (below drip pipes) and how it correlates to leaf gas exchange and water status in perennial crops (*e.g.* grapevine) are not clear.

We previously found that DRZ irrigation rate and not delivery depth was crucial to maintain grapevine water status and mitigate stress (Ma et al., 2019). In addition, we found that DRZ increased grape yields by 9–12% compared to traditional surface drip irrigation (SD) and that grapevine rooting was decreased in the top 60 cm soil profile suggesting that DRZ promoted deeper root growth (Ma et al., 2020). To provide further insight into root development of grapevines particularly in the deep soil profile and to measure the correlations between root growth, leaf gas exchange and whole-plant water status under DRZ compared to SD, a two-year field study (2017–2018) was conducted in a commercial vineyard in southcentral Washington, USA. Root distribution along the 0–160 cm soil profile was measured. Leaf gas exchange was monitored, and it was correlated to whole-plant water status which was measured through midday stem water potential (Ψ_stem-md_) and was recently reported (Ma et al., 2020). In addition, leaf area, as well as total carbon and nitrogen contents in leaves and shoots were measured to determine the differences in nutrient assimilation between SD and DRZ. We hypothesized that grapevines irrigated though DRZ have proportionally increased rooting at depth, concurrently with higher photosynthetic carbon assimilation rates which positively correlate to the diurnal plant water status.

## Materials and Methods

### Field Site Description

This study was conducted for two consecutive growing seasons (2017–2018) in a commercial vineyard of ten-year old, own-rooted *Vitis vinifera* L. cv. Cabernet Sauvignon in the Red Mountain American Viticultural Area (AVA) near Benton City, Washington (46°16′59″ N, 119°26′33″ W, 228 m a.s.l.). Mature and own-rooted Cabernet Sauvignon was selected as our experimental material because it is well adapted to irrigation and has been the top produced grape variety in Washington since 2015 with a high economic value (Washington State Wine Commission, USA, 2020). The vineyard rows were oriented north-south, with a spacing of 1.8 m and 2.5 m, respectively between vines and rows. A three-wire trellis was applied, and the vertical distances were 100 cm, 140 cm and 180 cm between the soil surface and each wire. Soil on our experimental vineyard was of the Aridisol order and classified as a Hezel loamy fine sand, consisting of 80% sand, 17% silt, and 3% clay along 0–40 cm profile (United States Department of Agriculture-Natural Resources Conservation Service, USA. Web Soil Survey, 2019), with 0.56% total carbon and 0.056% total nitrogen content based on soil chemical analysis. Daily precipitation and air temperature were recorded through an automated weather station operated by the Washington AgWeatherNet statewide system (AgWeatherNet, https://weather.wsu.edu/) located near the vineyard in Benton City, WA (approximately 1 km from the study site). Phenological stages of grapevines for all treatments were recorded based on the Biologische Bundesanstalt, Bundessortenamt und CHemische Industrie (BBCH) scale for bud break (stage 09), flowering (stage 65), fruit set (stage 71), veraison (stage 81) and harvest (stage 89) (Lorenz et al., 1995). To map the development of vines over the course of the study, all dates of phenological stage were reported as the average of all treatments (Pisciotta et al., 2018; Ma et al., 2019).

### Irrigation Systems and Experimental Design

Before all treatments started, all grapevines were drip-irrigated through a commercial SD system, which was installed at the same time of vineyard establishment. Another surface drip line was installed in each experimental row as part of tested DRZ and SD systems before the 2015 growing season. Grapevines had acclimated to both tested irrigation systems for two years (2015–2016) before conducting this two-year study. The vertical distance was 40 cm between the suspended drip line and bottom wire of the trellis and was 60 cm between the suspended drip line and the soil surface. Two pressure compensating emitters (CETA, Antelco, Longwood, FL, USA) were used by both irrigation systems for each tree and were located approximately 40 cm on either side of vine trunk, with a flow rate of 4 L h^−1^ vine^−1^. For the DRZ system, a hole with a diameter of 25.4 mm was drilled vertically to the 60 cm soil layer. The PVC tube (Schedule 40, 20 mm inner diameter) was cut into a length of 100 cm which was vertically inserted into the hole for water delivery, with a 40 cm length above ground. A PVC cap (Schedule 40, 21mm inner diameter) for each PVC tube was previously drilled to allow passage of feeder line, which connects the surface drip line with a drip emitter inside the tube. Details on designs and installation of SD and DRZ systems were described by Ma et al. (2020).

The experiment was implemented as a split-plot design in a randomized complete block design with three blocks. Irrigation rate was the whole-plot factor forming a complete block based on evapotranspiration for Cabernet Sauvignon (ETc = ETo × Kc) from bud break to harvest. The data for reference crop (grass) evapotranspiration (ETo) were collected through the same automated weather station mentioned above and were calculated using Penman-Monteith equation (Allen et al., 1998). The average crop coefficient (Kc = 0.5) was developed based on previous studies on wine grapes in southcentral Washington (Evans et al., 1993; Keller et al., 2016). Three levels of irrigation were applied from fruit set to harvest: high rate (0.75–0.80 ETc), moderate rate (0.60–0.65 ETc), and low rate (0.45–0.50 ETc). Irrigation amounts during different periods of phenological stages are shown in **Supplementary Table 1**. Irrigation method (either SD or DRZ) was the subplot factor and only one irrigation method was assigned to each subplot. Each subplot involved three contiguous rows with five vines in each row (3 rows × 5 vines = 15 vines per subplot), and measurements were only taken on the three central vines in the central row, with twelve buffer vines alongside to avoid unwarranted interference from neighboring treatments. All treatments (irrigation rate × irrigation method) were replicated three times.

### Irrigation Scheduling

All grapevines were irrigated from bud break to postharvest, with treatments implemented from fruit set to harvest in each year. Fertigation was implemented through surface drip lines (4 L h^−1^ vine^−1^) and was controlled by the vineyard manager. Liquid fertilizer (25% N, 0% P, 0% K, 3% S) was applied once for about 24 h between bud break and fruit set to avoid any interaction effects between irrigation method and fertilization, which was beyond the scope of this study. The irrigation interval (3-7 days) was determined by the vineyard manager based on weather, soil water content and long-standing guidelines to reach commercial production goals. Generally, vines were irrigated when soil water content in commercial plots (within 20 m to the boundary of treatment plots) decreased to 4 ± 1 mm, 11 ± 1 mm, and 12 ± 1 mm, respectively at a 20, 40, and 60 cm soil depth which were continuously monitored by EnviroSCAN probes (Sentek technologies, Australia). In the experimental site, two EnviroSCAN probes, respectively in SD- and DRZ-treated plots under the high irrigation rate, were used to monitor the changes in water content at 60 cm soil layer between late June and early July in 2017, which are presented in **Supplementary Figure 1**. Irrigation events for different treatment plots on the same day were started simultaneously. Battery powered controllers (11,000 L series, Galcon, Kfar Blum, Israel) were used for reducing the water amounts to designated rates, and actual amounts of applied water were recorded through small mechanical water meters (D.L. Jerman Co., Hackensack, NJ, USA) installed in each experimental row. After harvest, two more rounds of full irrigation, each for 24 h, were applied to refill soil moisture for helping grapevines prevent frost damage in winter. After that, no more irrigation was applied until the bud break of the following growing season.

### Leaf Gas Exchange

Measurements of leaf gas exchange were taken from fruit set to harvest in each growing season. One leaf (nodes 6–8 from the shoot tip) from each of the three central grapevines in each subplot was selected for leaf gas exchange measurement using the LCi-SD portable photosynthesis system (ADC BioScientific Ltd., Hoddesdon, UK). The broad leaf chamber was used with a window area of 6.25 cm^2^. Air flow rate was 200 ml min^−1^ and reference CO_2_ concentration was set at 400 µmol mol^−1^. Prior to taking measurements on a leaf, the chamber was closed and status of all the sensors inside the chamber were checked through readings on the display. Generally, the reading for ambient CO_2_ concentration should stabilize to give similar level of reference CO_2_ concentration. Readings for ambient H_2_O, photosynthetically active radiation (PAR) and chamber temperature should be also stable. Measurements were made after all of these checks were satisfactory. A portion of the leaf was enclosed in the chamber which took up to 2 min to stabilize its new microclimate inside the chamber and make readings. Leaves were mature, fully expanded, and exposed to sunlight. Net rate of CO_2_ assimilation (A), stomatal conductance (g_s_), transpiration rate (E) and intrinsic water use efficiency (WUE_i_, defined as the ratio between A and g_s_) were measured on sunny and clear days with incident PAR on leaf surface > 1,700 µmol m^−2^ s^−1^, typically right before the irrigation, between 10:00 am and 12:00 noon.

### *In Situ* Root Observation

One of the three central vines in the middle row of each subplot was selected for *in situ* root imaging, comprising three treatment replicates for analyses of root number (count tube^−1^) and root length density (RLD, mm cm^−2^). RLD was defined as the total root length per unit of root image area. Minirhizotron tubes (length × diameter = 180 cm × 6.35 cm) were installed at a distance of 30 cm from the vine trunk with an angle of 15° to the vine trunk, allowing observation of roots within a 0–160 cm soil profile. The exposed top of the root tubes (approximately 8-10 cm) was covered with aluminum tape and sealed with vinyl caps to avoid disturbance from light on root growth and to prevent light scattering and interference for imaging. Eight root images were taken at a dpi of 300 along the length of each tube using the CI-600 In Situ Root Imager (CID Bio-Science, Camas, WA, USA) operated by a tablet computer with CI-600 software installed (https://cid-inc.com/support/CI-600/software/). Root images were taken at phenological stages of fruit set, veraison, and harvest in each year. The size of each root image was 21.5 cm long and 19.6 cm wide, with approximately 0.8 cm overlap of adjacent images to guarantee the scan of the entire root area of interest. Root images were analyzed individually by using RootSnap! Image Analysis Software version 1.3.2.25 (CID Bio-Science, Camas, WA, USA). Details in operation of the root imager and root image analysis were described by Ma et al. (2019).

### Measurements of Leaf Area, Carbon and Nitrogen Contents in Leaves and Shoots

To help understand the leaf and shoot development of grapevines under DRZ, preliminary experiments were conducted at harvest in 2018 for investigating the influences of the DRZ on leaf size and carbon and nitrogen contents in leaves and shoots. Two mature leaves (one from the east side and another from the west side) on each grapevine were randomly selected for specific leaf area (SLA) measurement. Leaves were sampled by severing the petiole with a razor blade, then leaves from the same grapevine were put into a sampling bag and stored on ice. All samples were brought back to lab immediately for measuring leaf size by using the LI-3100C Area Meter (LI-COR Biosciences, NE, USA). After leaf size measurement, all leaf samples were put into an air-dryer at 60°C for at least 48 h. Dry samples were weighed for leaf biomass. SLA for each grapevine was calculated as:

$$\text{SLA}\;(\text{c}{\text{m}}^{2}{\text{g}}^{-1})=\frac{\text{leaf size}\;(cm^{2})}{\text{leaf biomass}\;(\text{g})}$$

Meanwhile, other sets of leaves and shoots were sampled for total carbon (C) and nitrogen (N) content analyses. Twelve leaves and twelve shoots from three central vines in the same subplot, with four leaves and four shoots collected per vine, were mixed and put into an air-dryer at 60°C for at least 48 h. All dried samples were milled into powder, and around 0.2 g powder per dry sample was sent for total carbon and nitrogen content analyses by using the TruSpec Micro analyzer (LECO Corp., MI, USA).

### Statistical Analyses

Data were analyzed separately by year. A two-way analysis of variance (ANOVA) adjusted for split-plot design was used to detect treatment effects on leaf gas exchange, root growth, leaf area, and total C and N contents, followed by Tukey’s HSD test as a post-hoc analysis for comparisons between different treatment groups. A one-way ANOVA was used to detect differences in total root number and RLD within each range of soil depth (20 cm intervals along 0–160 cm soil profile; 0–60 cm and 60–160 cm soil profiles) between two irrigation methods (SD and DRZ) at each irrigation rate. Statistical analyses were run by using R 3.4.3 statistical software package (www.r-project.org) at p-value = 0.05. Correlation analyses were performed to evaluate the strength of relationships between leaf gas exchange and Ψ_stem-md_ in grapevines. Linear equations and correlation coefficients were calculated with SigmaPlot 12.5 software (SPSS Inc., Chicago, IL, USA).

## Results

### Weather Trends

**Figure 1:**
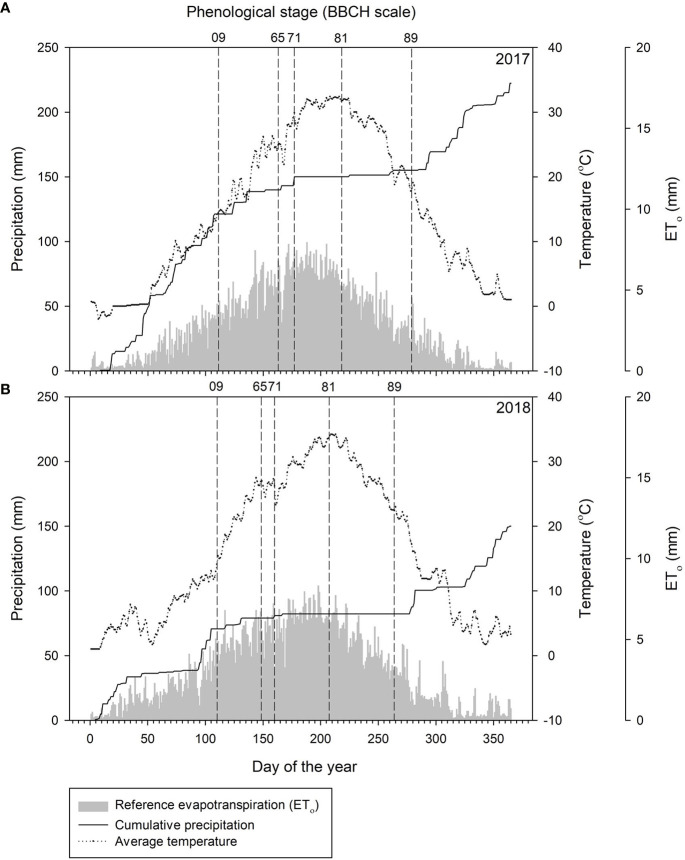
Cumulative daily precipitation (mm, solid lines), daily temperature (°C, dotted lines), daily reference evapotranspiration (ET_o_, mm, gray bars), and day of the year of phenological stage (dashed lines) in Red Mountain, WA, USA in **(A)** 2017 and **(B)** 2018. Weather data were collected from AgWeatherNet at Washington State University (http://weather.wsu.edu/). Phenological stages were based on BBCH scale for bud break (stage 09), flowering (stage 65), fruit set (stage 71), veraison (stage 81) and harvest (stage 89).

Weather patterns were different between the two years of the study (**Figure 1**). Total precipitation was 33% lower in 2018 (150.1 mm) than in 2017 (222.5 mm). Cumulative precipitation before bud break (Stage 09) and between bud break and fruit set (Stage 71) were 42% and 64% lower, respectively in 2018 than in 2017. Precipitation was extremely limited from fruit set to harvest (Stage 89) in both years and was similar after harvest. Annual temperature was 1.2°C higher in 2018 as compared to 2017. Although average temperature before bud break was 2°C higher in 2018 than in 2017, average temperatures near bud break (14 days prior to bud break) were similar between years. Between the stages of bud break and flowering, the average temperature was 1.4°C higher in 2018 than in 2017. The annual reference evapotranspiration (ET_o_) was higher in 2018 (1155.5 mm) than in 2017 (1064.8 mm); however, accumulated ET_o_ from bud break to harvest was similar between years.

### Leaf Gas Exchange

**Figure 2:**
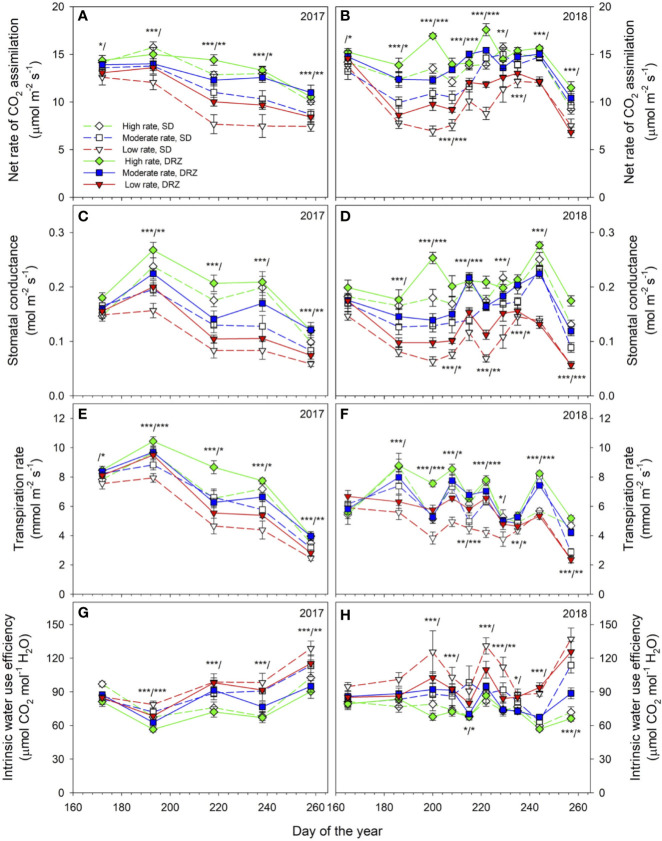
Leaf gas exchange under surface drip (SD, dashed lines) and direct root-zone (DRZ, solid lines) irrigation in 2017–2108. **(A, B)**: net CO_2_ assimilation rate (A, µmol m^−2^ s^−1^); **(C, D)**: stomatal conductance (g_s_, mol m^−2^ s^−1^); **(E, F)**: transpiration rate (E, mmol m^−2^ s^−1^); and **(G, H)**: intrinsic water use efficiency (WUE_i_, µmol CO_2_ mol^−1^ H_2_O). Three irrigation rates were set based on crop evapotranspiration (ET_c_) for Cabernet Sauvignon: Diamonds with green lines, squares with blue lines, and triangles with red lines represent irrigation rates at high (0.75–0.80 ET_c_), moderate (0.60–0.65 ET_c_) and low (0.45–0.50 ET_c_), respectively. Asterisks on the left side of the slash indicate statistically significant differences between effects of irrigation rate, and ones on the right side of the slash indicate statistically significant differences between effects of irrigation method. *, ** and *** represent statistical differences at P ≤ 0.05, 0.01 and 0.001, respectively. Error bars represent standard error (n = 9).

**Figure 3:**
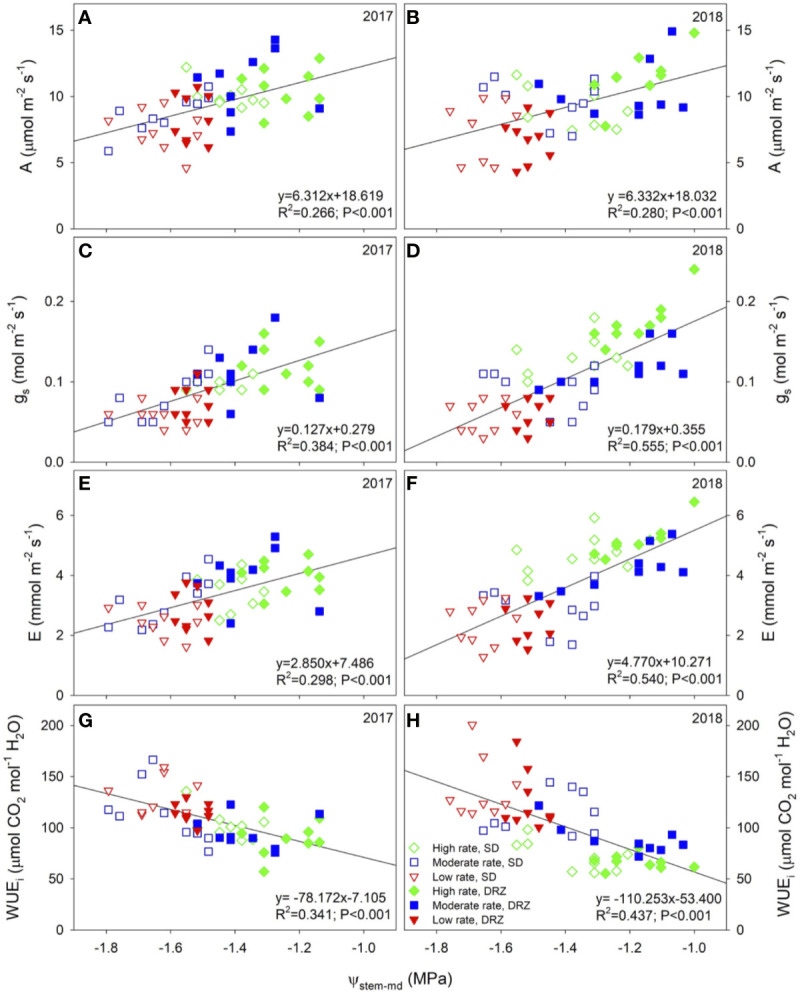
Linear correlations between leaf gas exchange and midday stem water potential (Ψ_stem-md_, MPa) in 2017 and 2018. Positive correlations were found between **(A, B)** Ψ_stem-md_ and net CO_2_ assimilation rate (A, µmol m^−2^ s^−1^), **(C, D)** Ψ_stem-md_ and stomatal conductance (g_s_, mol m^−2^ s^−1^), and **(E, F)** Ψ_stem-md_ and transpiration rate (E, mmol m^−2^ s^−1^). Negative correlation was found between **(G, H)** Ψ_stem-md_ and intrinsic water use efficiency (WUE_i_, µmol CO_2_ mol^−1^ H_2_O). Open symbols represent the surface drip irrigation (SD), and closed symbols represent the direct root-zone irrigation (DRZ). Three irrigation rates were set based on crop evapotranspiration (ET_c_) for Cabernet Sauvignon: Green diamonds, blue squares, and red triangles represent irrigation rates at high (0.75–0.80 ET_c_), moderate (0.60–0.65 ET_c_) and low (0.45–0.50 ET_c_), respectively. R^2^: coefficient of determination. Data were from measurements on Day of the year 258 and 257, respectively in 2017 and 2018 and were pooled within each year (n = 54).

Both irrigation rate and method significantly influenced leaf gas exchange in both years (**Figure 2**). In general, grapevines irrigated through DRZ had higher rate of net CO_2_ assimilation (A), accompanied by higher rates of stomatal conductance (g_s_) and transpiration (E) (**Figures 2A–F**). Meanwhile, decreases in irrigation rate reduced A, g_s_, and E (**Figures 2A–F**). Intrinsic water use efficiency (WUE_i_) had opposite relationships with irrigation rate and method compared to the other three parameters, as decreases in irrigation rate as well as DRZ strategy improved WUE_i_ (**Figures 2G, H**). The most significant differences between treatment effects were found during the hottest time of each growing season, usually from mid-July to early September. On average, DRZ significantly improved A, g_s_ and E by 16–24, 16–32, and 12–30%, respectively during those periods. Significant linear correlations (P<0.001) were found between leaf gas exchange and midday stem water potential (Ψ_stem-md_) during late growing season in both years, as higher Ψ_stem-md_ was accompanied by increased A, g_s_, E and decreased WUE_i_. Correlations were the strongest at harvest compared to other phenological stages within each year and were stronger in 2018 than in 2017 (**Figure 3**).

### Root Distribution

**Figure 4:**
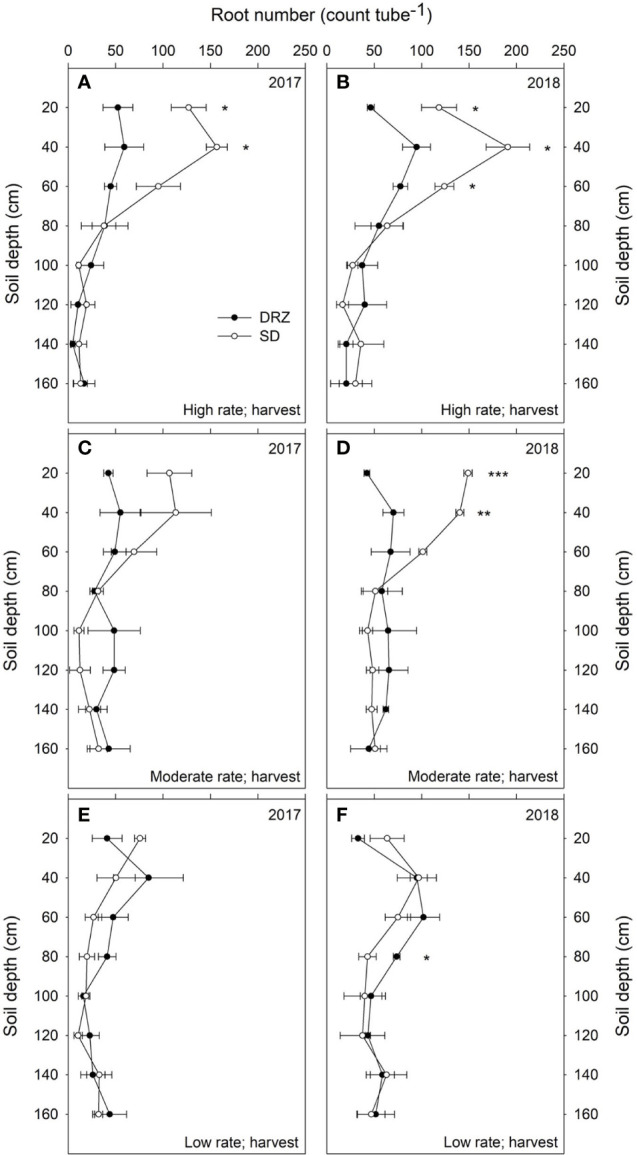
Total root number (count tube^−1^) along the 0-160 cm soil profile under surface drip (SD, open circles) and direct root-zone (DRZ, closed circles) irrigation at harvest in 2017 and 2018. Three irrigation rates were set based on crop evapotranspiration (ET_c_) for Cabernet Sauvignon: **(A, B)** high rate: 0.75–0.80 ET_c_; **(C, D)** moderate rate: 0.60–0.65 ET_c_; and **(E, F)** low rate: 0.45–0.50 ET_c_. *, ** and *** represent statistical differences at P ≤ 0.05, 0.01 and 0.001, respectively. Error bars represent standard error (n=3).

Treatment effects on root number and RLD were found in both years. Compared to SD, irrigation through the DRZ system significantly reduced total root number in the 0–160 cm soil profile by 20% at fruit set (right after treatments were applied) and by 23% at version in 2017. Similar patterns were also found at fruit set (11% decrease) and veraison (16% decrease) in 2018, although those decreases were not statistically significant. Decreases in irrigation rate from high to low significantly reduced total root number in the 0–160 cm soil profile for grapevines irrigated through the SD system; however, no significant reduction was found in grapevines irrigated through the DRZ system. When the whole soil profile (0–160 cm) was considered, differences in irrigation rate and method showed no significant effects on RLD over the course of this study; however, higher total root number and RLD were found in 60–160 cm soils under DRZ than under SD at the moderate rate, with significant differences found in 2017 (**Supplementary Figures 2** and **3**). Comparisons of root number and RLD between SD and DRZ within 20 cm intervals along the 0–160 cm soil profile at fruit set and veraison are shown in **Supplementary Figures 4–7**. No significant increases in total root number and RLD under DRZ were found within each of 20 cm intervals along 60–160 cm soil profile across all irrigation rates except the low rate at harvest in 2018, where grapevines irrigated through DRZ had significantly higher root number at 60–80 cm soil depth (**Figure 4**).

**Figure 5:**
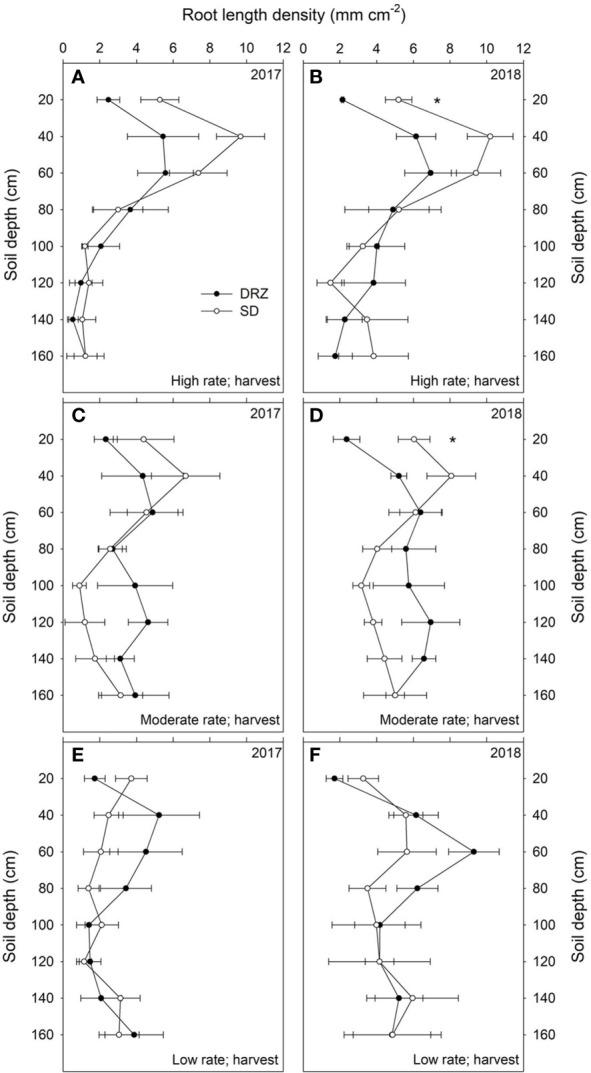
Root length density (RLD, mm cm^−2^) along the 0–160 cm soil profile under surface drip (SD, open circles) and direct root-zone (DRZ, closed circles) irrigation at harvest in 2017 and 2018. Three irrigation rates were set based on crop evapotranspiration (ET_c_) for Cabernet Sauvignon: **(A, B)** high rate: 0.75–0.80 ET_c_; **(C, D)** moderate rate: 0.60–0.65 ET_c_; and **(E, F)** low rate: 0.45–0.50 ET_c_. * represents statistical differences at P < 0.05, 0.01 and 0.001, respectively. Error bars represent standard error (n = 3).

However, the most significant treatment effects both on root number and RLD were found within 0–60 cm soil profile. DRZ significantly reduced total root number in top 60 cm soil at high and moderate irrigation rates in both years, with 50–60% less total root number for grapevines under DRZ compared to SD, but no significant decrease was found at low irrigation rate (**Supplementary Figure 2**). For grapevines irrigated though SD, decreased irrigation rate from high to low significantly reduced total root number in the 0–60 cm soil profile, as 60 and 46% fewer roots were found at harvest in 2017 and 2018, respectively (**Figure 4**). However, grapevines under DRZ showed no significant differences in total root number in the 0–60 cm soil profile between high and low irrigation rates in both years (**Figure 4**). Compared to SD, DRZ also reduced root length density (RLD) by 30–40% in the upper 60 cm soil at high and moderate irrigation rates, but no decrease was detected at low irrigation rate in both years (**Supplementary Figure 3**). More specifically, significant differences in RLD between irrigation methods were found in the top 0–20 cm soil profile, as DRZ reduced RLD by 50–60% compared to SD at high and moderate rates in 2018 (**Figure 5**).

### Specific Leaf Area, Total Carbon and Nitrogen Contents in Leaves and Shoots

Specific leaf area (SLA) was slightly higher for grapevines irrigated through DRZ than SD within each irrigation rate, especially at the low rate, where 5.3% higher SLA was found (**Supplementary Figure 8**). Decreases in irrigation rates from high to moderate and from high to low reduced SLA by 4.4 and 8.7%, respectively, with a 3.3% more reduction from high to low rate for grapevines irrigated through SD compared to DRZ (**Supplementary Figure 8**). Significant differences in total N content and C:N ratio in leaves were found between the two irrigation methods, as 8.4% higher total N content and 6.5% lower C:N ratio in leaves were found with adoption of DRZ (**Table 1**). No significant differences in total C content in leaves and total C and N contents in shoots were found.

**Table 1:** Mean total carbon (C) percentage, total nitrogen (N) percentage and carbon to nitrogen ratio (C:N ratio) in leaves and shoots as influenced by irrigation rate and method using two-way analysis of variance (ANOVA).

Main effect		Leaf			Shoot		
		C (%)	N (%)	C:N ratio	C (%)	N (%)	C:N ratio
**Irrigation rate**							
High		46.07	1.47	31.43	48.88	0.45	109.75
Moderate		46.89	1.52	30.93	49.07	0.47	105.82
Low		46.84	1.47	32.01	48.90	0.47	103.24
**Irrigation method**							
SD		46.27	1.43^b^	32.51^a^	48.95	0.46	107.87
DRZ		46.92	1.55^a^	30.41^b^	48.96	0.47	104.66
**ANOVA**							
Rate		n.s.	n.s.	n.s.	n.s.	n.s.	n.s.
Method		n.s.	*	*	n.s.	n.s.	n.s.
Rate × Method		n.s.	n.s.	n.s.	n.s.	n.s.	n.s.

n.s., not significant at P > 0.1.

*, **, *** significant difference at P ≤ 0.05, 0.01 and 0.001, respectively.

Within each year, means of main factors followed by different letters are significantly different at P ≤ 0.05 according to the Tukey’s HSD test.

## Discussion

Given current climate change scenarios, it has become evident that more efficient use of sparse water resources is of paramount importance for viticulture sustainability (Medrano et al., 2015; Fraga et al., 2018). Many perennial crops such as grapevines, almonds, apples, and pomegranates, in particular rely on supplemental irrigation to maintain growth and yield during periods of drought stress (Romero et al., 2004; Zhang et al., 2017; Pisciotta et al., 2018; Zhong et al., 2019). While there has been a positive shift from less sustainable methods of irrigation such as spray or furrow irrigation to more sustainable drip irrigation methods, improvements are still needed to enhance water productivity (Ayars et al., 2015). Direct root-zone irrigation (DRZ) was introduced recently as an efficient subsurface drip irrigation strategy to improve water use efficiency and sustain grape production in semi-arid regions (Ma et al., 2019). This study further advanced our knowledge of the novel irrigation strategy by investigating the eco-physiological responses of Cabernet Sauvignon grapevines to the DRZ and found improved leaf gas exchange with deep root development compared to the traditional surface drip irrigation (SD). These findings will help guide efficient grape cultivation in semi-arid climates with DRZ which could be also adopted in other agroecosystems.

This study found significant improvements in leaf gas exchange of grapevines under DRZ compared to SD in both years with different weather patterns. Those improvements were possibly attributed to higher water availability within the root zone under DRZ from fruit set to harvest, as the higher soil water content was detected in the 60 cm soil layer where the water was delivered (**Supplementary Figure 1**). Al-Omran et al. (2005) also found increased water content in soil layers treated by subsurface drip irrigation which benefitted crop performance in sandy soil. Delivering water through subsurface irrigation systems could provide more water for crop growth by reducing soil water evaporation (Ayars et al., 1999), thus grapevines irrigated through DRZ had more stomata that remained open for gas exchange and experienced less diurnal water stress, which was indicated by higher midday stem water potential (Ψ_stem-md_) compared to SD with a progress of water deficit (Ma et al., 2020). Influences of irrigation method on leaf gas exchange were amplified in the summer with limited precipitation, higher temperature and reference evapotranspiration (ET_o_), as they induce the stomatal closure and reduction of plant water potential (Limousin et al., 2013), thus the soil water availability became a major limiting factor that significantly influenced the photosynthetic capacity of grapevines. Compared to the wet growing season of 2017, decreased cumulative precipitation accompanied by increased average temperature and ET_o_ in 2018 probably reduced soil water availability for grapevine growth, which intensified water stress in grapevines as revealed by decreased Ψ_stem-md_ across all treatments (Ma et al., 2020). Therefore, improvements in leaf gas exchange for grapevines under DRZ were more significant in 2018 than in 2017. Due to a major role of water availability on grapevine growth in arid climates, influences of irrigation rate on leaf gas exchange were consistently significant from fruit set to harvest in both years, which are in accordance with previous studies (Chaves, 2004; Costa et al., 2007; Keller et al., 2016). These findings indicate that a precise regulation of soil water content through DRZ is vital for optimizing the leaf CO_2_ assimilation of grapevines to cope with heat- and drought-induced adversities in semi-arid climates.

Observation of root growth showed increased total number and root length density (RLD) of deep roots (60–160 cm soil layers) compared to shallow roots (0–60 cm soil layers) at moderate and low irrigation rates than at high rate, revealing that deeper root systems in grapevines could be developed through regulated deficit irrigation as proposed in previous studies (Dry et al., 2000; Costa et al., 2007; Romero et al., 2012). Recent studies observed the water uptake patterns of grapevine and other plant species in semi-arid regions, and found that with progressed water stress a large proportion of water was derived from deep soils (Wang et al., 2017; Savi et al., 2019). Deeper root distributions have been detected for grapevines with increased drought tolerance (Smart et al., 2006; Fort et al., 2017), indicating that deep root systems may access groundwater in deeper soil to maintain higher leaf photosynthetic rate and to relieve plant water stress, which is consistent with our findings (Ma et al., 2019; Ma et al., 2020).

Significant differences in root distribution between DRZ and SD were also found as the irrigation method influences plant rooting patterns (Bassoi et al., 2003; Romero et al., 2004). In this study, DRZ affected root growth by adjusting the water availability in different soil layers. DRZ significantly reduced root development in the topsoil, partly due to limited irrigation water that was available in surface soil, which is consistent with previous studies on root distribution of different crops under subsurface drip irrigation (Phene et al., 1991; Machado et al., 2003; Romero et al., 2004; Al-Omran et al., 2005; Kong et al., 2012; Pisciotta et al., 2018). Moreover, higher root number and RLD were found under DRZ compared to SD within 60-160 cm soil profile, indicating that DRZ possibly increases root growth by improving water availability below 60 cm soil depth. Differences in root distribution might also exist below 160 cm soil depth, although the majority of grapevine roots (e.g. >80%) are reported to be in the upper 100 cm of soil in managed agricultural systems (Smart et al., 2006). However, this study could not make any further conclusions. A portion of roots might remain near the subsurface emitters, as significantly higher root number was found under DRZ within 60–120 cm soil depth, where they can easily access irrigation water (Romero et al., 2004). Although the eco-physiological responses of grapevine roots to DRZ emphasized in the current study indicate the changes in soil water availability, one caveat is that direct observations of the soil water distribution along the entire soil profile are limited. Future studies will better elucidate the relationship between deep root development and soil moisture distribution in response to DRZ.

Soil type and texture could be another important factor influencing the impacts of DRZ on grapevines. Thus far, the DRZ system has been tested only in sandy soil, which is a highly permeable soil type. Although significant effects were found, this soil type possibly compromises the positive influences of DRZ on improvement in soil water availability due to its lower water holding capacity compared to clay soils. Thus, more significant treatment effects may be detected in a less permeable soil type, similar to the findings of Al-Omran et al. (2005) where soil moisture content was increased by adding clay deposits. Future research should focus on a comprehensive comparison of soil water content between different soil types.

Reduced water availability to some extent restricts root ability for water and nutrient uptake, which might lower the nutrient concentration, such as carbon (C) and nitrogen (N), in leaves and shoots for regulating plant development (Saud et al., 2017). In our study, no earlier basal leaf abscission occurred between veraison and harvest in both years, and no significant differences in total C and N contents between different irrigation rates were found in leaves and shoots, indicating that no extreme water stress was reached that could severely hamper the grapevine growth. However, grapevines irrigated through DRZ experienced less water stress, which was also indicated by higher leaf N content, lower C:N ratio in mature leaves and slightly higher specific leaf area in 2018. These patterns are consistent with findings reported previously in cowpea and sorghum(Anyia and Herzog, 2004; Chen et al., 2015). In this scenario, grapevine possibly invested a greater portion of resources to accelerate aboveground growth rather than root development. Instead of producing new roots for improvement in aboveground growth, DRZ might also elongate the root lifespan for water and nutrient uptake through increased soil water availability (McCormack and Guo, 2014). Those aspects are also worth investigating in future studies.

## Conclusion

This study found higher photosynthetic carbon assimilation rates in grapevines irrigated through the direct root-zone irrigation (DRZ) compared to surface drip irrigation (SD) and provided insights into rooting patterns under subsurface irrigation with seasonal drought. Grapevine alters rooting patterns under DRZ by significantly restricting shallow root growth and encouraging root development in the deeper soil profile. Deep rooting patterns could help grapevines take water from deeper layers for optimizing grapevine growth and alleviating water stress during periods of heat and drought. Future studies need to investigate the relationship between grapevine rooting patterns and dynamics of soil water distribution in different soil types and in different grapevine varieties to help them better adapt to arid climates.

## Data Availability Statement

The datasets supporting the conclusion of this article will be made available by the authors, without undue reservation, to any qualified researcher.

## Author Contributions

XM, PJ, and KS designed and supervised the research. XM performed the research and analyzed the data. XM drafted the manuscript. PJ and KS critically revised the manuscript and verified quality of written English. All authors contributed to the article and approved the submitted version.

## Funding

This research was supported by USDA Western Sustainable Agriculture Research and Education Program Graduate Student Grant (GW17-058); Washington State Department of Agriculture Specialty Crop Block Program Project (K1768); Washington State Grape and Wine Research Program (Nos. 3019-3818; 3019-6818); Northwest Center for Small Fruit Research (No. 2072-21000-047-16); USDA National Institute of Food and Agriculture, Hatch project (No. 1014527).

## Conflict of Interest

The authors declare that the research was conducted in the absence of any commercial or financial relationships that could be construed as a potential conflict of interest.
