# Supplementary material for: Improving Net Photosynthetic Rate and Rooting Depth of Grapevines Through a Novel Irrigation Strategy in a Semi-Arid Climate
Source: Front Plant Sci. 2020 Aug 27;11:575303. doi: 10.3389/fpls.2020.575303 (PMC7481399; doi:10.3389/fpls.2020.575303)
Supplement: Supplementary file 1 [file Image_1.pdf]

Supplementary Material

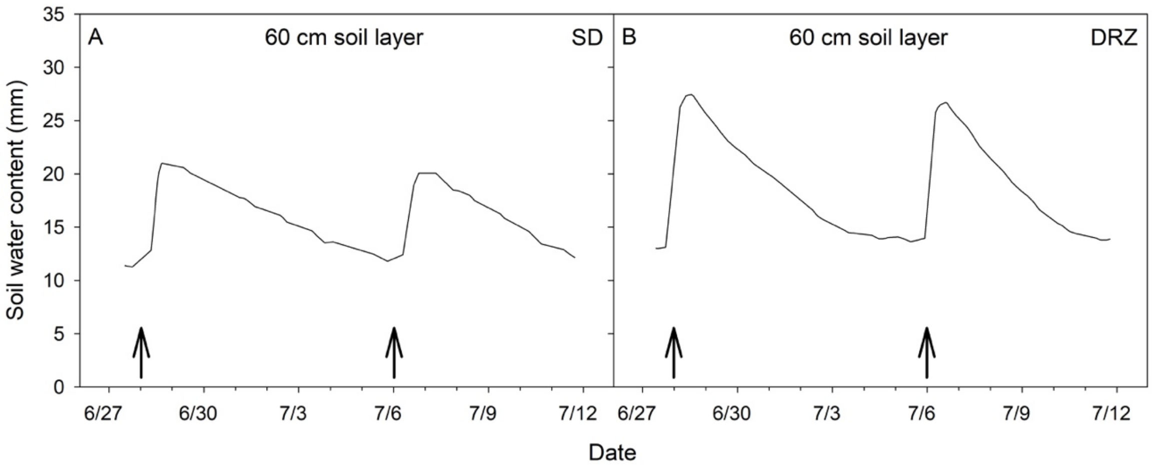

Supplementary Figure 1. Changes in soil water content (mm) at 60 cm depth of soil under **(A)** surface drip (SD) and **(B)** direct root-zone (DRZ) irrigation at the high rate (0.75-0.80 crop evapotranspiration) between late June and early July in 2017. Arrows represent irrigation events with the same amount of water applied during each event.

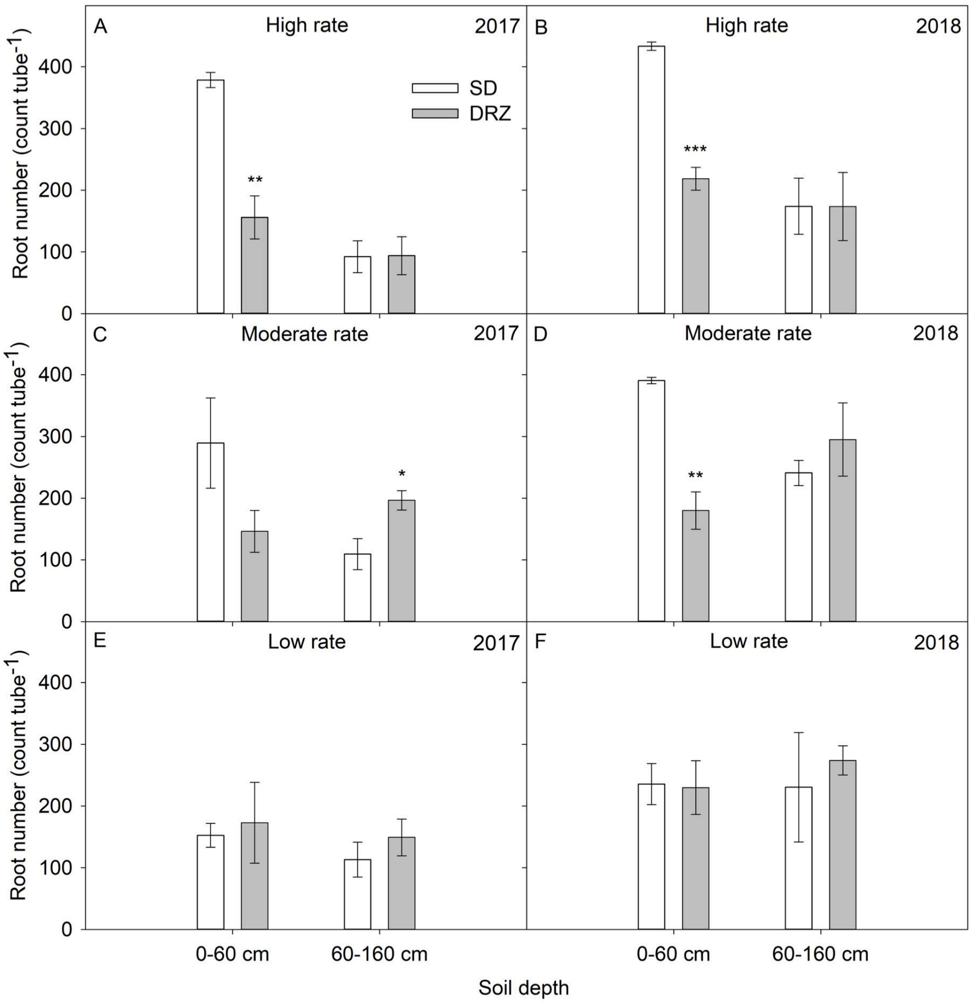

Supplementary Figure 2. Total root number (count tube^{-1}) along the 0-60 cm, and the 60-160 cm soil profiles under surface drip (SD) and direct root-zone (DRZ) irrigation. Three irrigation rates were set based on crop evapotranspiration (ET_c) for Cabernet Sauvignon: **(A-B)** high rate: 0.75-0.80 ET_c; **(C-D)** moderate rate: 0.60- 0.65 ET_c; and **(E-F)** low rate: 0.45-0.50 ET_c. Data were compared within each range of soil depth, *, ** and *** represent statistical differences at $P \leqslant 0.05$, 0.01 and 0.001, respectively. Data were collected at harvest in 2017 and 2018, and error bars represent standard error (n=3).

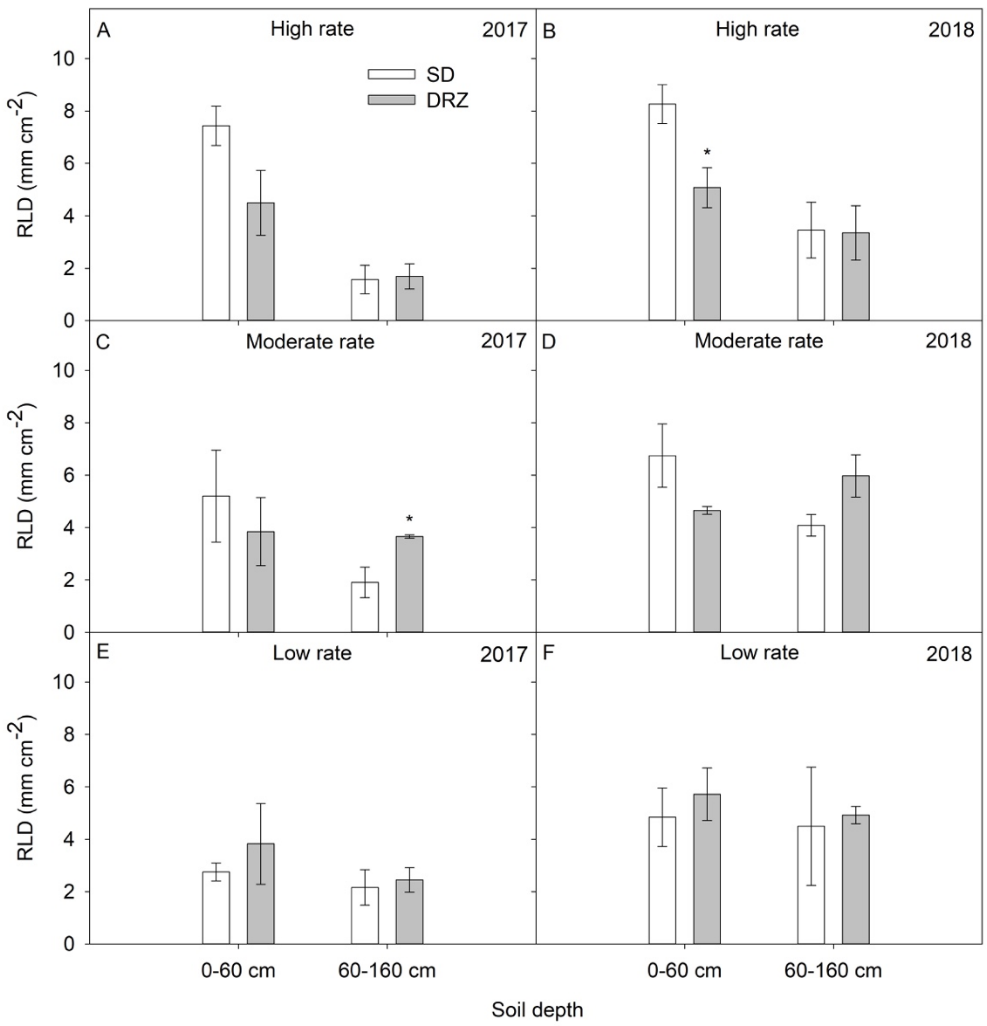

Supplementary Figure 3. Root length density (RLD, mm cm^{-2}) along the 0-60 cm, and the 60-160 cm soil profiles under surface drip (SD) and direct root-zone (DRZ) irrigation. Three irrigation rates were set based on crop evapotranspiration (ET_c) for Cabernet Sauvignon: **(A-B)** high rate: 0.75-0.80 ET_c; **(C-D)** moderate rate: 0.60- 0.65 ET_c; and **(E-F)** low rate: 0.45-0.50 ET_c. Data were compared within each range of soil depth, * represents statistical differences at $P \leqslant 0.05$. Data were collected at harvest in 2017 and 2018, and error bars represent standard error (n=3).

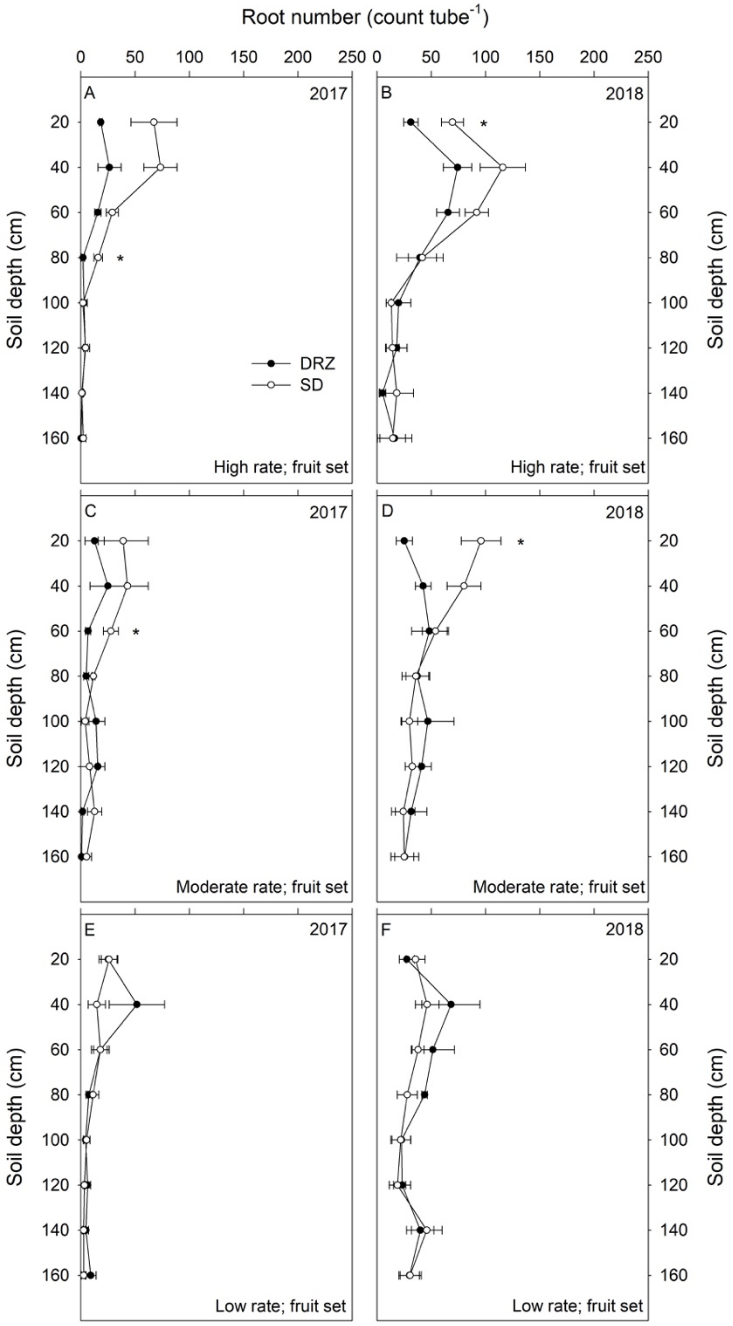

Supplementary Figure 4. Total root number (count tube^{-1}) along the 0-160 cm soil profile under surface drip (SD, open circles) and direct root-zone (DRZ, closed circles) irrigation at fruit set in 2017 and 2018. Three irrigation rates were set based on crop evapotranspiration (ET_c) for Cabernet Sauvignon: **(A-B)** high rate: 0.75-0.80 ET_c; **(C-D)** moderate rate: 0.60- 0.65 ET_c; and **(E-F)** low rate: 0.45-0.50 ET_c. * represents statistical differences at $P \leqslant 0.05$. Error bars represent standard error (n=3).

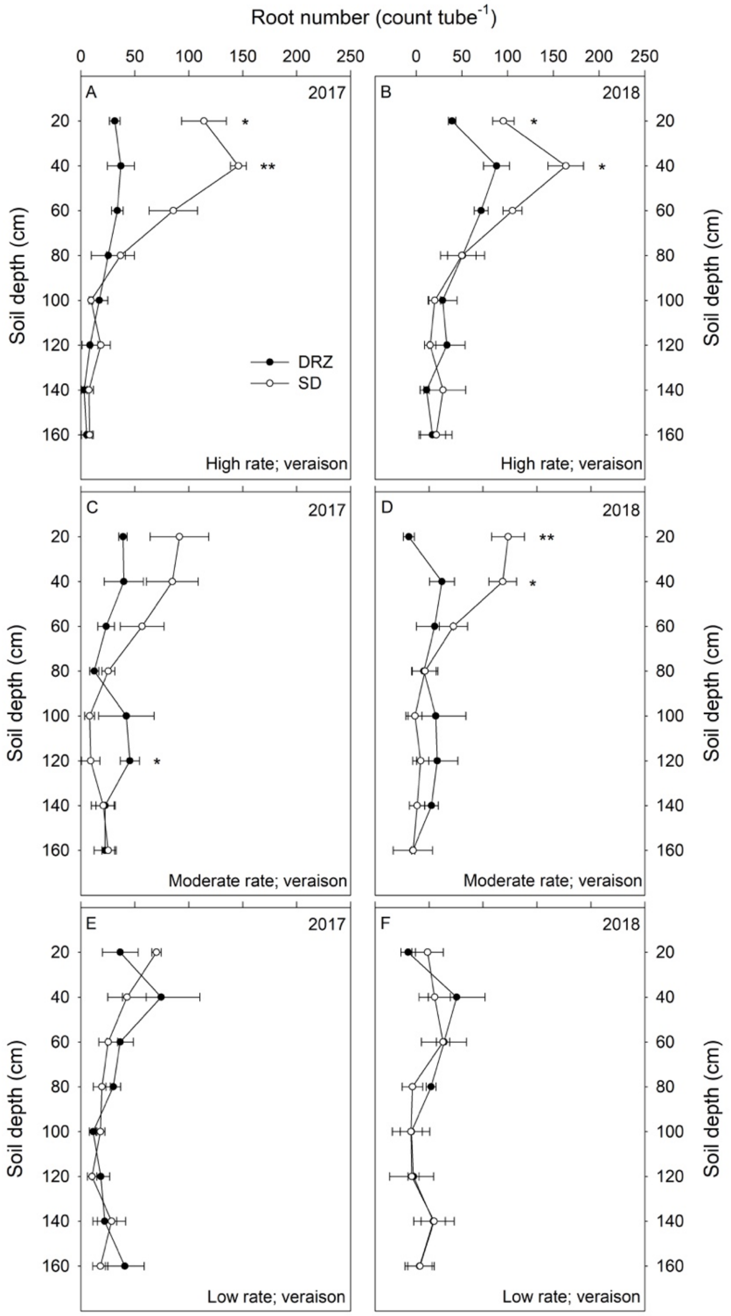

Supplementary Figure 5. Total root number (count tube^{-1}) along the 0-160 cm soil profile under surface drip (SD, open circles) and direct root-zone (DRZ, closed circles) irrigation at veraison in 2017 and 2018. Three irrigation rates were set based on crop evapotranspiration (ET_c) for Cabernet Sauvignon: **(A-B)** high rate: 0.75-0.80 ET_c; **(C-D)** moderate rate: 0.60- 0.65 ET_c; and **(E-F)** low rate: 0.45-0.50 ET_c. * and ** represent statistical differences at P≤0.05 and 0.01, respectively. Error bars represent standard error (n=3).

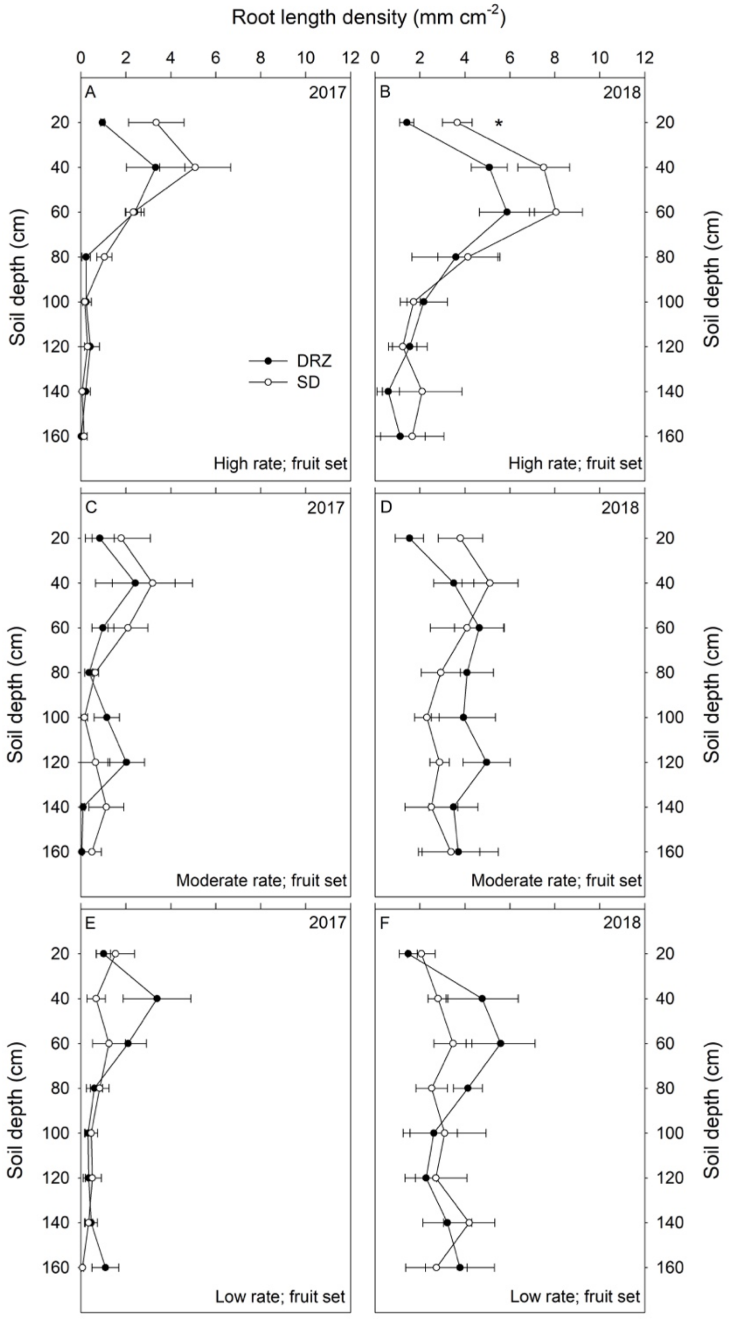

Supplementary Figure 6. Root length density (RLD, mm cm^{-2}) along the 0-160 cm soil profile under surface drip (SD, open circles) and direct root-zone (DRZ, closed circles) irrigation at fruit set in 2017 and 2018. Three irrigation rates were set based on crop evapotranspiration (ET_c) for Cabernet Sauvignon: **(A-B)** high rate: 0.75-0.80 ET_c; **(C-D)** moderate rate: 0.60- 0.65 ET_c; and **(E-F)** low rate: 0.45-0.50 ET_c. * represents statistical differences at P≤0.05. Error bars represent standard error (n=3).

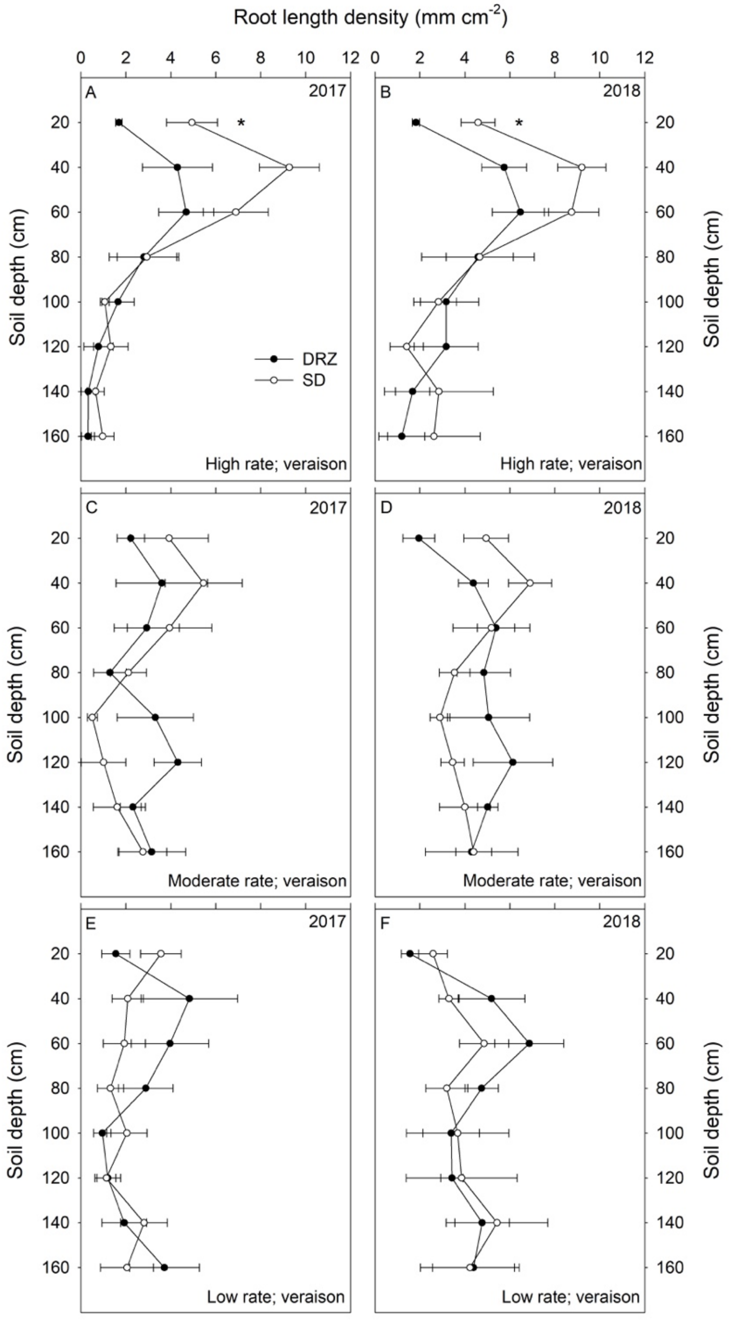

Supplementary Figure 7. Root length density (RLD, mm cm^{-2}) along the 0-160 cm soil profile under surface drip (SD, open circles) and direct root-zone (DRZ, closed circles) irrigation at veraison in 2017 and 2018. Three irrigation rates were set based on crop evapotranspiration (ET_c) for Cabernet Sauvignon: **(A-B)** high rate: 0.75-0.80 ET_c; **(C-D)** moderate rate: 0.60- 0.65 ET_c; and **(E-F)** low rate: 0.45-0.50 ET_c. * represents statistical differences at $P \leqslant 0.05$. Error bars represent standard error (n=3).

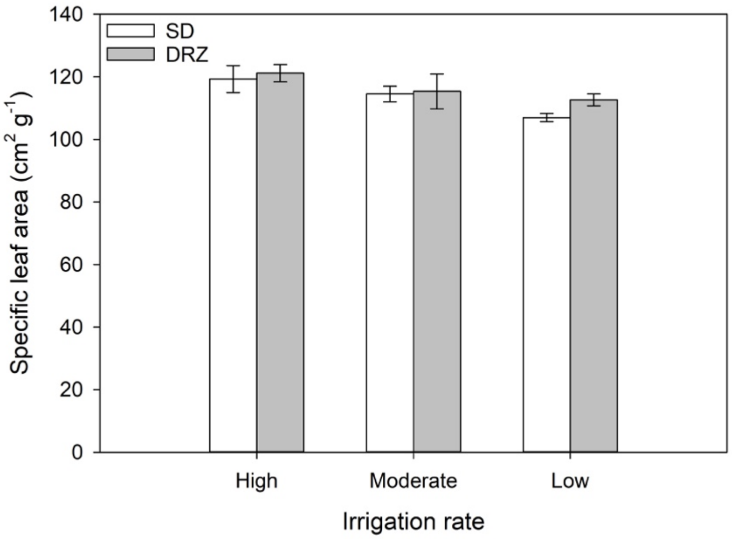

Supplementary Figure 8. Specific leaf area (cm^2 g^{-1}) at harvest under surface drip (SD) and direct root-zone (DRZ) irrigation within each of irrigation rates. Three irrigation rates were set based on crop evapotranspiration (ET_c) for Cabernet Sauvignon: high rate: 0.75-0.80 ET_c; moderate rate: 0.60-0.65 ET_c; and low rate: 0.45-0.50 ET_c. Data were collected at harvest in 2018, and error bars represent standard error (n=9).

Supplementary Table 1. Irrigation amounts (mm) during different phenological stages in 2017-2018.

Year	Irrigation rate	Bud break to fruit set	Fruit set to veraison	Veraison to harvest	Postharvest	Total
2017	High	83.3	104.7	104.7	27.8	320.5
	Moderate	83.3	76.1	76.1	27.8	263.3
	Low	83.3	46.8	46.8	27.8	204.7
2018	High	53.9	122.9	122.9	27.0	326.7
	Moderate	53.9	91.0	91.0	27.0	262.9
	Low	53.9	58.3	58.3	27.0	197.4

Vines in the same row received the same amount of irrigation water. Same amounts of irrigation water were applied to each vine from bud break to fruit set and postharvest within each year. Deficit irrigation was applied through our treatments from fruit set to harvest in each year. Actual irrigated water amounts were recorded by mechanical water meters in each row.
